# Effect of linex treatment on IFN-γ and IL-4 in mice infected with Trichinella

**DOI:** 10.1186/s12879-024-10202-9

**Published:** 2024-11-28

**Authors:** Shimaa Attia Atta, Zeinab H. Fahmy, Eman A.H. Selim, Tarek Aboushousha, Reham Refaat Mostafa

**Affiliations:** 1 Department of Immunology, Theodor Bilharz Research Institute, Cairo, Egypt; 2 Department of Parasitology, Theodor Bilharz Research Institute, Cairo, Egypt; 3 Department of Pathology, Theodor Bilharz Research Institute, Cairo, Egypt; 4 Departments of Medical Parasitology Faculty of Medicine, Cairo University, Cairo, Egypt

**Keywords:** Trichinellosis, Probiotic, Linex, IFN-γ, IL-4, IL-13

## Abstract

Trichinellosis is a zoonotic, foodborne parasitic infection causing muscle damage. This study investigated the potential therapeutic effects of the commercially available probiotic treatment Linex, both alone and in combination with Albendazole (ALB), on the intestinal and muscular stages of Trichinella spiralis infection in mice, assessing outcomes through parasitological, immunological, and histopathological measures. This study is the first to demonstrate the synergistic effect of combining the commercially available probiotic Linex with Albendazole for trichinellosis treatment. By enhancing both parasitological and immunological outcomes, this combined therapy not only significantly reduces parasite burden but also modulates the immune response, shifting it toward a protective Th1 profile. In parasitological terms, the highest adult and larval count reduction was observed in combined Linex and Albendazole treatment (100%, 97.7%) respectively. Lesser percentage of reduction were recorded in Linex alone therapy (43.2%, 88.4%) respectively. Histopathologically there was amelioration of the inflammatory cellular infiltration in all treated groups with best results in combined Linex and Albendazole treatment. Immunologically, serum IFN-γ levels increased significantly in all treated groups with highest levels in combined Linex and Albendazole treatment, while IL-4 and IL-13 level decreased significantly in all treated groups with best results observed in Linex alone treatment. To conclude; combined Linex and Albendazole treatment of mice infected with *T. spirals* could ameliorate the infection and improve the immune response.

## Background

Trichinella spiralis is a widely distributed nematodal infection, infecting a wide range of mammalian hosts [1]. The pathology of trichinellosis begins with an initial inflammatory response during the intestinal phase, and is followed by both inflammatory and allergic responses as the larvae invade the host’s muscles [2]. Intestinal infection characteristically induces T-helper- 1 cell (Th1) cytokines early in infection followed by a gradual increase cytokines released by T-helper 2 cells such as IL-4 and IL-13, which play a crucial roles in the expulsion of the worms [3].

Treatment of trichinellosis is challenging, Mebendazole and Albendazole are the most commonly used medications for treating trichinellosis [4], however, their effectiveness varies depending on the stage of infection, in addition to the adverse effects associated with Albendazole such as hepatotoxicity, gastrointestinal disturbances, and potential teratogenic effects [5]. Additionally, growing concerns about drug resistance have been reported, as Trichinella strains are increasingly able to survive conventional treatments, particularly during the muscle phase of the infection [6] therefore, securing new drugs or drug combinations has arisen to be more safer, and more effective treatment options and also could minimize the side effect.

Probiotics seems to be a promising approach that deal with many clinical conditions such as parasitic infestation.

Many species of probiotics are documented to be beneficial to humans the main two genera are *Lactobacillus* and *Bifidobactrium*, each of them include many species [7], however, it is clear that probiotic properties are specific to both the strain and the tissue type, meaning that the impact of probiotics can vary across different human tissues [8]. But they have been proven to have strong anti-inflammatory and immunomodulatory action [9], in addition to their antiparasitic effect [10], probiotics have been demonstrated to improve intestinal mucosal barriers, reduce inflammation, and modulate both innate and adaptive immune responses, by increasing IFN-γ production, which is essential for combating the infection during the intestinal phase, and affect T-helper cell Interleukins production during muscle phase. In addition to its effect on the immune system it competes with pathogens for adhesion sites within the gastrointestinal tract, thereby reducing the parasite’s ability to establish an infection making probiotics a promising adjuvant therapy in infections such as trichinellosis [11, 41].

## Materials and methods

### Experimental animals

Experiments were conducted on 60 mice, with the sample size calculated based on a power analysis to ensure the detection of significant differences between groups, as outlined by Charan [12, 13]. The mice were obtained from the Animal House at TBRI, Giza, Egypt. Mice were laboratory-bred for 6 to 8 week, weighing 18 gm.

### Experimental design

Sixty mice were randomly divided into 4 groups with 6 mice in each subgroup.

- Group I (Drug control): Non infected treated with Linex including 6 mice.
- Group II (Healthy control) including 6 mice.
- Group III (intestinal phase of infection) mice infected with Trichinella spiralis receive treatment first day post infection (pi) and sacrificed 7 days pi. This group subdivided into:

Group IIIa: Infected untreated including 6 mice.

Group IIIb: Infected treated with Linex including 6 mice.

Group IIIc: Infected treated with ALB including 6 mice.

Group IIId: Infected treated with Linex + ALB including 6 mice.

- Group IV (muscle phase of infection): mice receive treatment 30 day pi and sacrificed after 35 days pi.

This group subdivided into:

Group IVa: Infected untreated including 6 mice

Group IVb: Infected treated with Linex including 6 mice.

Group IVc: Infected treated with ALB including 6 mice.

Group IVd: Infected treated with Linex + ALB including 6 mice.

### Infection

larvae of Trichinella spiralis species were obtained by artificial digestion of tissues using 1%pepsin and 1%HCl at 37 °C for 4 h following the Kapel and Gamble protocol [12, 13] then stored in saline solution. The larvae were sieved and repeatedly sedimented in distilled water for washing, and then counted using a cell counting chamber. Each mouse was infected with 12,500 larvae, following the protocol outlined by Munoz and Cole [14].

### Therapeutic agents

Albendazole was purchased commercially (Albendazole ^R^ 400 pharma care) and given by oral inoculation via oral gavage in dose of 50 mg/kg for 5 days [15].

Probiotic was purchased commercially (linex^R^ Adult Sandoz), also given by oral inoculation via oral gavage in dose of 15 mg/kg for 3 days.

Linex^R^ Adult are hard capsules each contain Lactobacillus Acidophilus and Bifidobacterium lactis BB-12.

Both treatments were started either on day 1 post-infection for the intestinal phase group or on day 30 post-infection for the muscle phase group.

### Euthanasia

Intraperitoneal anesthesia was used for mice euthanasia. They were administered an anesthetic-anticoagulant solution (500 mg/kg thiopental and 100 units/mL heparin) via intraperitoneal injection [16].

Mice in Group III (intestinal phase of infection) were sacrificed 7 days post-infection (pi), while mice in Group I, II (healthy and drug control respectively) and IV (muscle phase of infection) were sacrificed 35 days post-infection.

### Parasitological examination

#### Counting the adult worm in intestine

5 cm long pieces of small intestine were cut and placed in a sieve and incubated overnight in 0.9% saline at 37 °C. After incubation, the gut samples were taken out, and the worms in the sediment were counted using a stereomicroscope at 40× magnification [17].

#### Counting the larvae in muscles

The excised diaphragm was weighed and then digested with pepsin-HCl to release the larvae, following this, the larvae were counted using a microscope at 40×magnification. According to García et al. [18], the larval load was determined by calculating the total number of larvae per gram of muscle. The percent reduction in the larval load was calculated among different treated groups.

This pepsin-HCl artificial digestion according to Wojtkowiak-Giera et al. [19] is the method of choice for larval counting in muscle.

### Histopathological examination

Diaphragm, tongue, and skeletal muscle samples were fixed in 10% neutral buffered formalin for 24 h then processed through ascending grades of ethyl alcohol (70%, 90%, 100%), followed by xylene, and finally embedded in wax to create formalin-fixed paraffin-embedded blocks. Section 4 μm thick were cut and then stained with Hematoxylin and Eosin (H&E) for evaluation where ten low power microscopic fields (10x) were examined for larval capsules count and Inflammatory reaction intensity was scored as mild take score 1, moderate with score 2 and intense reaction with score 3 [20].

### Immunological tests and cytokine profile

Blood samples were collected from each mouse of different groups and left to clot at room temperature. Serum samples were collected after centrifuging clotted blood at 2500 rpm for 20 min, then aliquoted and stored at -20 °C for later use. Serum concentration of mouse IFNγ, IL-4 and IL-13 was assesed using an ELISA kit (Cat.No EL0026Mo, HS-EL0110Mo and EL0094Mo respectively (SUNLONG, China). In this assay; the test utilizes the double antibody sandwich ELISA detection technique. Specific anti-mouse antibodies were precoated on high-affinity ELISA micro well plates. Standards and test samples were added to the wells of the ELISA plate. After incubation, either IFNγ, IL-4 or IL-13 present in the samples binds to the solid-phase antibodies. After washing to remove unbound components, biotinylated detection antibodies are added and incubated. After washing to remove unbound biotinylated antibodies, streptavidin-HRP labeled with horseradish peroxidase was added. After washing, addition of TMB substrate solution to each well take place. Only the wells containing the required Ag exhibited a blue color, which turned yellow after addition of the stop solution. At a wavelength of 450 nm the optical density (OD) of the samples was assessed using an automated ELISA reader (Biorad, USA). OD values, which are proportional to the concentration of the cytokine, were calculated from the plotted standard curve.

### Statistical analysis

Quantitative values of the measured parameters were expressed as mean ± standard deviation (SD). Data were analyzed by one way-ANOVA to determine significance of differences between studied groups using Statistical Package for Social Sciences (SPSS), version 16.0. All statistical tests were considered significant at *p* < 0.05.

## Results

### Parasitological assessment of Trichinella infection

#### A-reduction in adult worm load

**Fig. 1:**
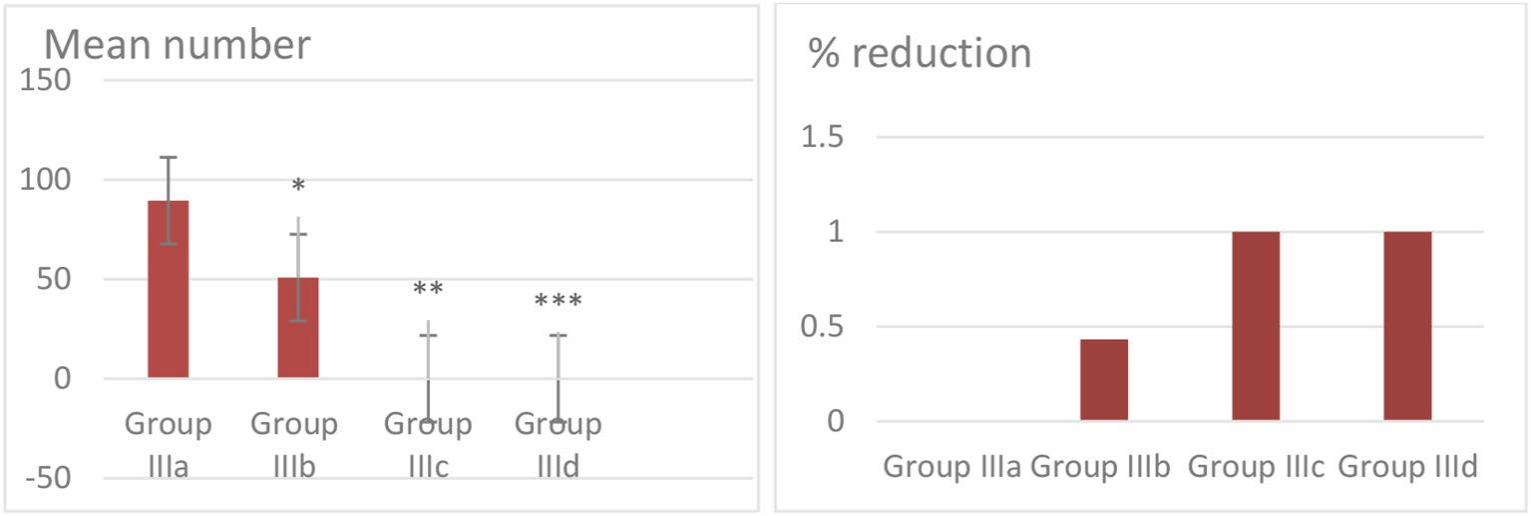
Mean adult worms numbers in the intestine and % reduction of them in different treated groups

No Adult Trichinella were found in either the non-infected treated group (group I) or the healthy control group (group II), Table 1 Fig. 1 displays the mean number of adult worms detected in the intestines of mice across both the infected untreated group and all the infected treated groups in the form of mean ± SD and the percent reduction of adult worm in all treated groups.

The Albendazole-treated group (IIIc) and the combined Albendazole and Linex-treated group (IIId) demonstrated a 100% reduction in the number of worms compared to the infected untreated group (*p* < 0.05) (indicating that these treatments were equally effective in eliminating adult Trichinella. In contrast, the Linex-only treated group (IIIb) showed a mean number of adult worms 50.83 ± 2.3* with significant reduction by 43.2% (*p* < 0.05) indicating a moderate therapeutic effect of Linex when used alone.

**Table 1:** Mean adult worm numbers in the intestine and percent reduction across different treatment groups

Group	Mean Adult Worm Count (± SD)	% Reduction
**Group IIIa**: Infected untreated	89.5 ± 2.9	0%
**Group IIIb**: Infected, treated with Linex	50.83 ± 2.3*	43.2%
**Group IIIc**: Infected, treated with Albendazole	0**	100%
**Group IIId**: Infected, treated with Linex + Albendazole	0***	100%

*Group IIIb (infected, treated with Linex) shows a significant difference compared to the infected untreated group (*p* < 0.05)

**Group IIIc (infected, treated with Albendazole) shows a significant difference compared to infected untreated group (*p* < 0.05)

***Group IIId (infected, treated with Linex + Albendazole) shows a significant difference compared to infected untreated group (*p* < 0.05)

#### B-Reduction in muscle larval load

**Fig. 2:**
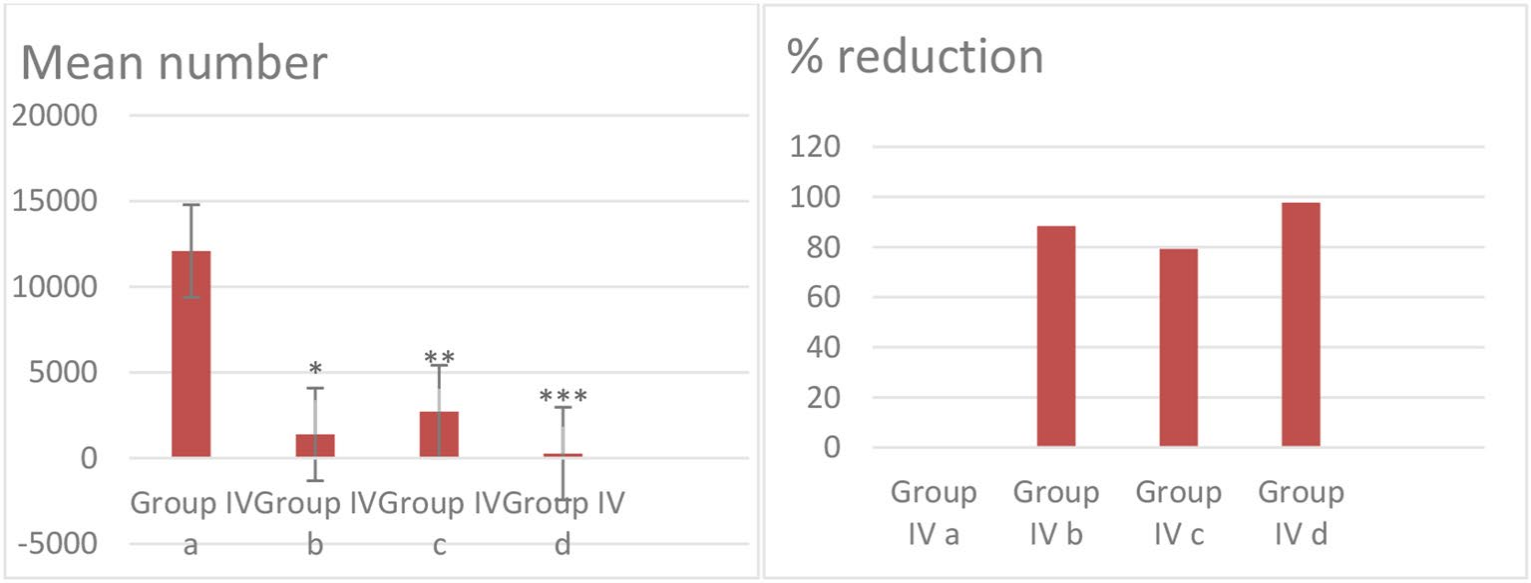
Mean number of larvae per gram of muscle beside % reduction of larvae in different drug treated groups

No larvae were found in the diaphragm, tongue, and skeletal muscles samples of the non-infected drug-treated group (group I) or the healthy control group (group II). Table 2 Fig. 2 present the mean number of larvae per gram of muscle ± SD and the percent reduction in both the infected untreated group and all the infected treated groups.

Albendazol and Linex combination group (IVd) show mean number of larvae 266.0***±124.7 with significant percent reduction of 97.7% (*p* < 0.05) while Linex only treated group (IVb) show least percent reduction of 88.4% and mean number of larvae of1383.3*± 134.4 (*p* < 0.05). Albendazole only treated group (IVc) reduce the larval load significantly by 79.2% and mean number of larvae of 2716.2**±279.4 (*p* < 0.05).

**Table 2:** Mean number of larva in the muscle and percent reduction across different treatment groups

Group	Mean number of larvae/gm. of muscle (± SD)	% Reduction
**Group IVa**: Infected untreated	11916.67	0
**Group IVb**: Infected, treated with Linex	1383.3 ± 134.4*	88.4%
**Group IVc**: Infected, treated with Albendazole	2716.2 ± 279.4**	79.2%
**Group IVd**: Infected, treated with Linex + Albendazole	266.0 ± 124.7***	97.7%

*Group IVb (infected, treated with Linex) shows a significant difference compared to the infected untreated group (*p* < 0.05)

**Group IVc (infected, treated with Albendazole) shows a significant difference compared to infected untreated group (*p* < 0.05)

***Group IVd (infected, treated with Linex + Albendazole) shows a significant difference compared to infected untreated group (*p* < 0.05)

### Histopathological changes among intestinal and muscle tissues

There was a significant decrease observed in all groups compared to the control in the inflammatory intensity score in intestine. The small intestine show normal villous architecture in the drug control group (group I) and healthy control group (group II), also the treated groups with Albendazole only and combined Albendazole and Linex (groups IIIc, IIId) show almost normal villous architecture, while in infection control group (group IIIa) there is Intense cellular infiltration consisting of lymphocytes and plasma cells with distorted villous pattern and Trichinella spiralis larva also Linex only treated group (group IIIb) show moderate cellular infiltration of plasma cells and lymphocytes and distorted villous pattern where Trichinella spiralis larva is present.

Compared to Albendazole alone which show moderate cellular infiltration addition of Linex to Albendazole decrease the inflammatory infiltration, with the best tissue recovery and most significant larval/cyst degeneration.

There was significant decreased in the Score of the inflammatory intensity in muscle in all groups as compared to control.

In drug control group (group I) and the healthy control group (group II) the muscle appears normal with regular muscle bundles and nuclei located peripherally, separated by a thin fibrous stroma.

The infected non treated group IVa show an intact Trichinella spiralis cyst with intact capsule and larva also show intense inflammatory reactions in form of plasma cells, histiocytes and lymphocytes among infected muscle fiber and in the pericapsular area of encysted larvae.

**Fig. 3:**
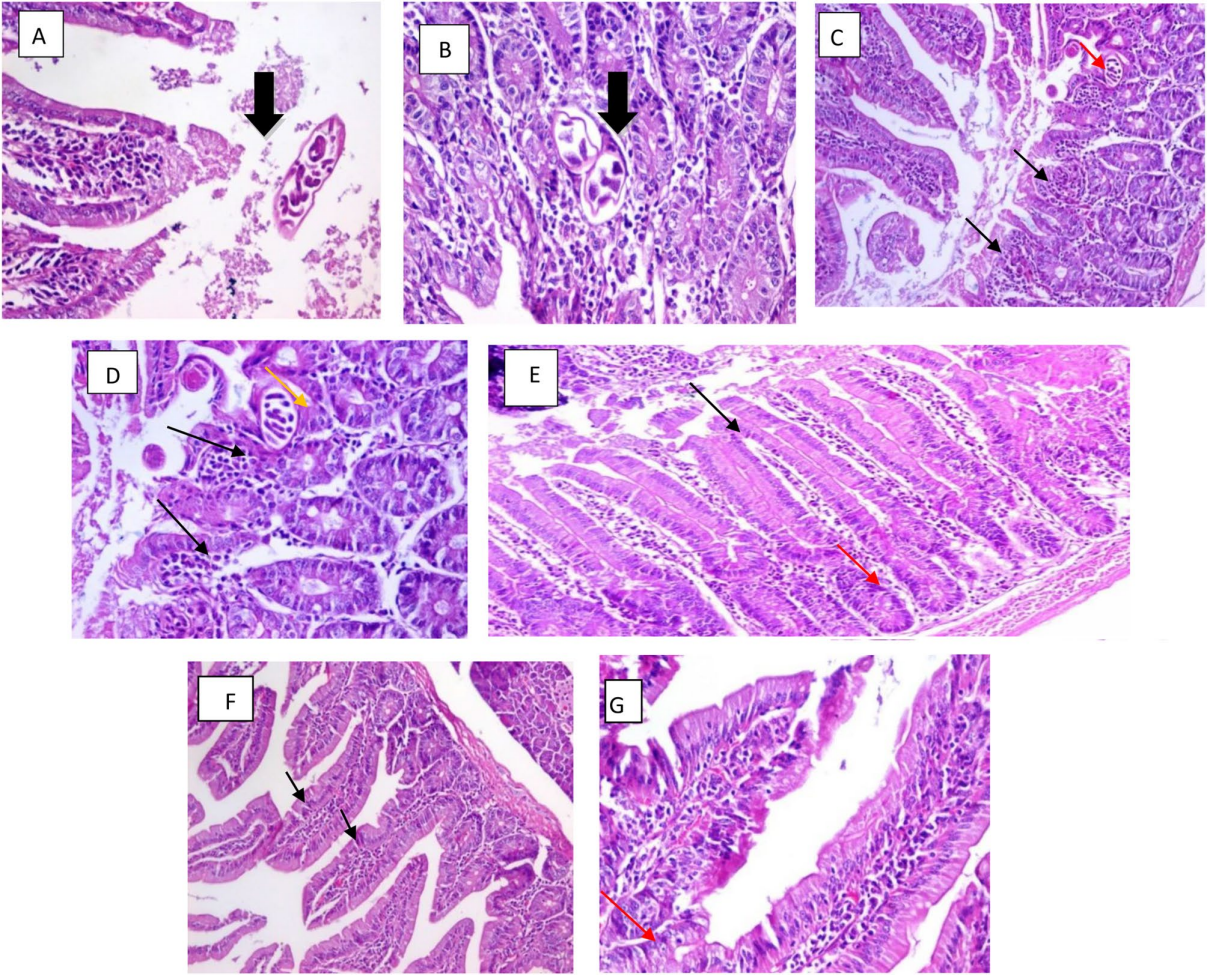
(**A**, **B**) Sections in the intestine of infection control group (IIIa) showing the larvae of T.S in the lumen and within the intestinal mucosa (black arrow) (Hematoxylin and eosin stain, ×200). (**C**, **D**) the intestine of Linex only treated group (IIIb) there is distorted villous pattern (black arrows) and larvae of T.S. (red arrow) with moderate cellular infiltration (yellow arrow) (Hematoxylin and eosin stain, ×100 & ×400). (**E**) Intestine of Albendazole only treated group (IIIc) showing a normal villous pattern (black arrows) (Hematoxylin and eosin stain, ×200) moderate inflammatory cellular infiltration within the villous core (red arrow). (**F**, **G**) Section in the intestine of combined Albendazole and Linex treated group (IIId) showing A normal villous pattern (black arrows) (Hematoxylin and eosin stain, ×100), and mild inflammatory cellular infiltration within the villous core (red arrow) (Hematoxylin and eosin stain, ×400)

**Fig. 4:**
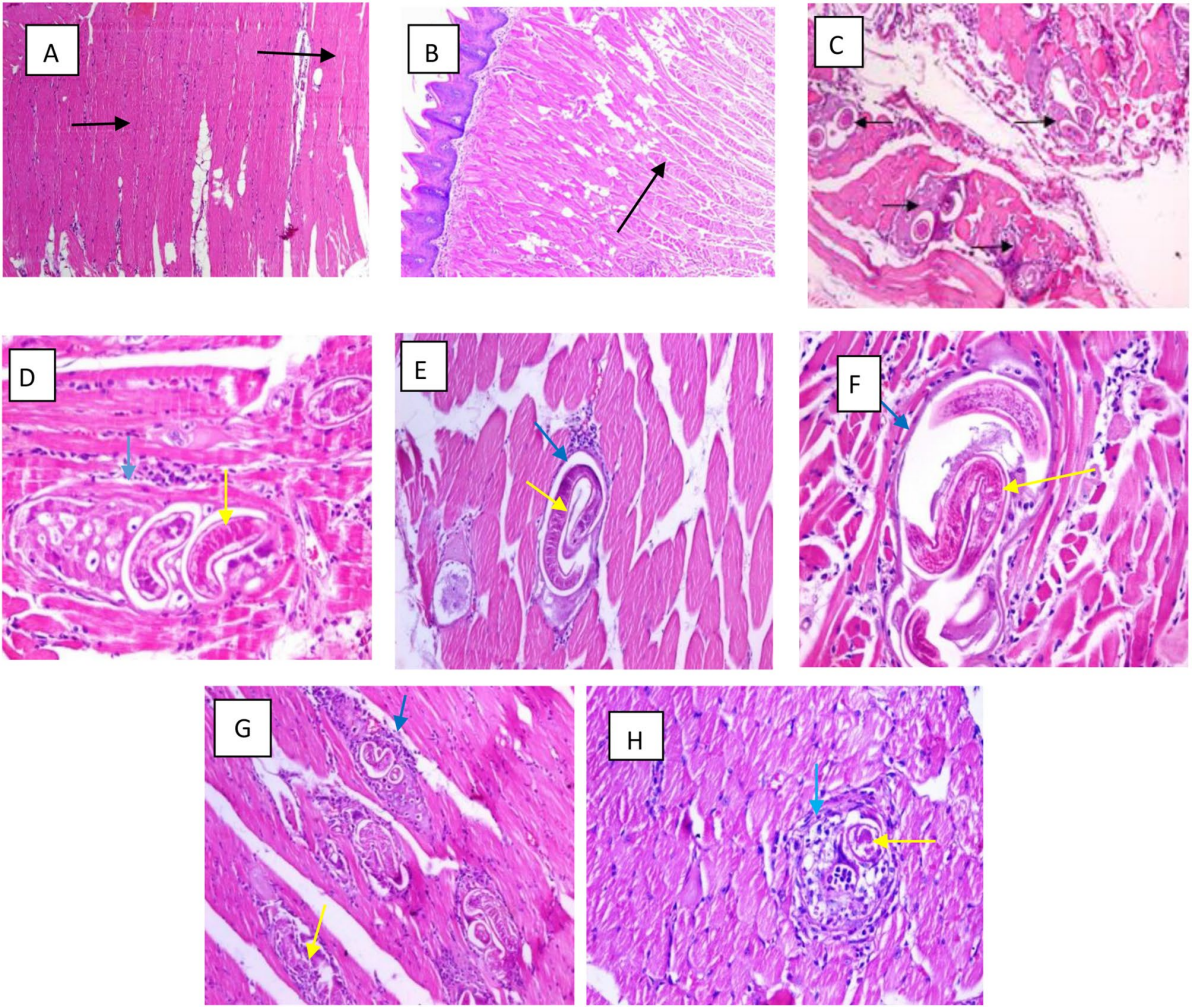
(**A**, **B**) Section in the skeletal muscles of the normal control group (I) and drug control group (II) respectively both show a well-organized, intact muscle fibers The fibers are elongated, multinucleated, and show clear striations (black arrow), with no evidence of cysts, inflammation, or tissue damage (Hematoxylin and eosin stain, ×100). (**C**) Section in the skeletal muscles of infected control group showing many T.S cysts (black arrows) (Hematoxylin and eosin stain, ×100). (**D**) Showing an intact T.S. cyst with intact capsule (blue arrow) and larva (yellow arrow) with intense inflammatory reaction (Hematoxylin and eosin stain, ×200). (**E**, **F**) The skeletal muscle of Linex only treated group showing a large swollen T.S. cyst with thin capsule (blue arrow) and intact larva (yellow arrow) with moderate inflammatory cellular infiltration (Hematoxylin and eosin stain, ×400). (**G**) Section in the skeletal muscles of Albendazole only treated group showing few T.S. cysts with focally degenerated capsule (blue arrow) and larve (yellow arrow) with moderate cellular infiltration (Hematoxylin and eosin stain, ×200). (**H**) Section in the skeletal muscle of combined Albendazole and Linex treated group showing a T.S. cyst with compeletly degenerated capsule (blue arrow) and larva (yellow arrow) and mild inflammatory cellular infiltrate (Hematoxylin and eosin stain, ×400)

Combined Albendazole and Linex treated group (group IVd) show the best histopathological findings, inflammatory infiltration was minimal, and the surrounding muscle tissue appeared near normal, Trichinella spiralis cyst show compelete degeneration in the capsule and larva. Albendazole only treated group (IVc) there was a noticeable decrease in the number of Trichinella cysts, many of which showed focal degeneration of the capsule and larvae, inflammatory infiltration was moderate, though less severe than in the Linex-only group (IVb) which show large swollen Trichinella spiralis cyst with thin capsule and intact larva, Linex only group affect the inflammatory intensity as well to adequate extent with moderate inflammatory cellular infiltration Figs. 3 and 4 show the histopatological changes among the healthy and drug control groups (I, II) and infection control groups during intestinal phase (IIIa) and muscular phase of infection (Iva). and treated groups with different treatment regimen during intestinal phase (IIIb, IIIc, IIId) and muscular phase of infection (IVb, IVc, IVd).

### Cytokine profile in treated groups

Analysis of serum samples of the muscular phase showed comparable results regarding cytokine levels.

Analysis of serum samples of the muscular phase showed comparable results regarding cytokine levels.

Treatment with either Linex, Albendazole or both increased INFγ levels, however the combined therapy had the highest effect on INFγ levels in comparison to the infected group (*p* > 0.05) 105.05***±0.096 and 44.11 ± 0.107 respectively followed by the Albendazole that showed significant elevation in comparison to the infective control group (*p* > 0.05) 73.03**±0.138 and finally comes the Linex that also showed a significant elevation in comparison to the infective control group 51.95*±0.171 (*p* > 0.05).

Regarding IL-4; with treatment with Linex, Albendazole, or combined therapy; the levels decreased in compared to infection control group. The lower significant levels were found in the group treated with Linex alone (33.16***±0.298) in comparison to infected group (56.10 ± 0.115) (*p* > 0.05) followed by the combined Albendazole and Linex treated group (41.16**±0.472) (*p* > 0.05) and finally treatment with Albendazole alone which decrease IL-4 with significance in comparison to the infected control group (47.61*±3.363) (*p* > 0.05).

Treatment with Linex, Albendazole, or combined therapy show significant decrease in the levels of IL-13. Linex alone could decrease IL-13 levels the most in comparison to the infected control group (41.92***±0.134 and 60.74 ± 0.526 respectively) (*p* > 0.05) followed by combined Albendazole and Linex therapy (45.93**±0.149) (*p* > 0.05) and finally Albendazole alone (52.95*±0.171) (*p* > 0.05).

**Fig. 5:**
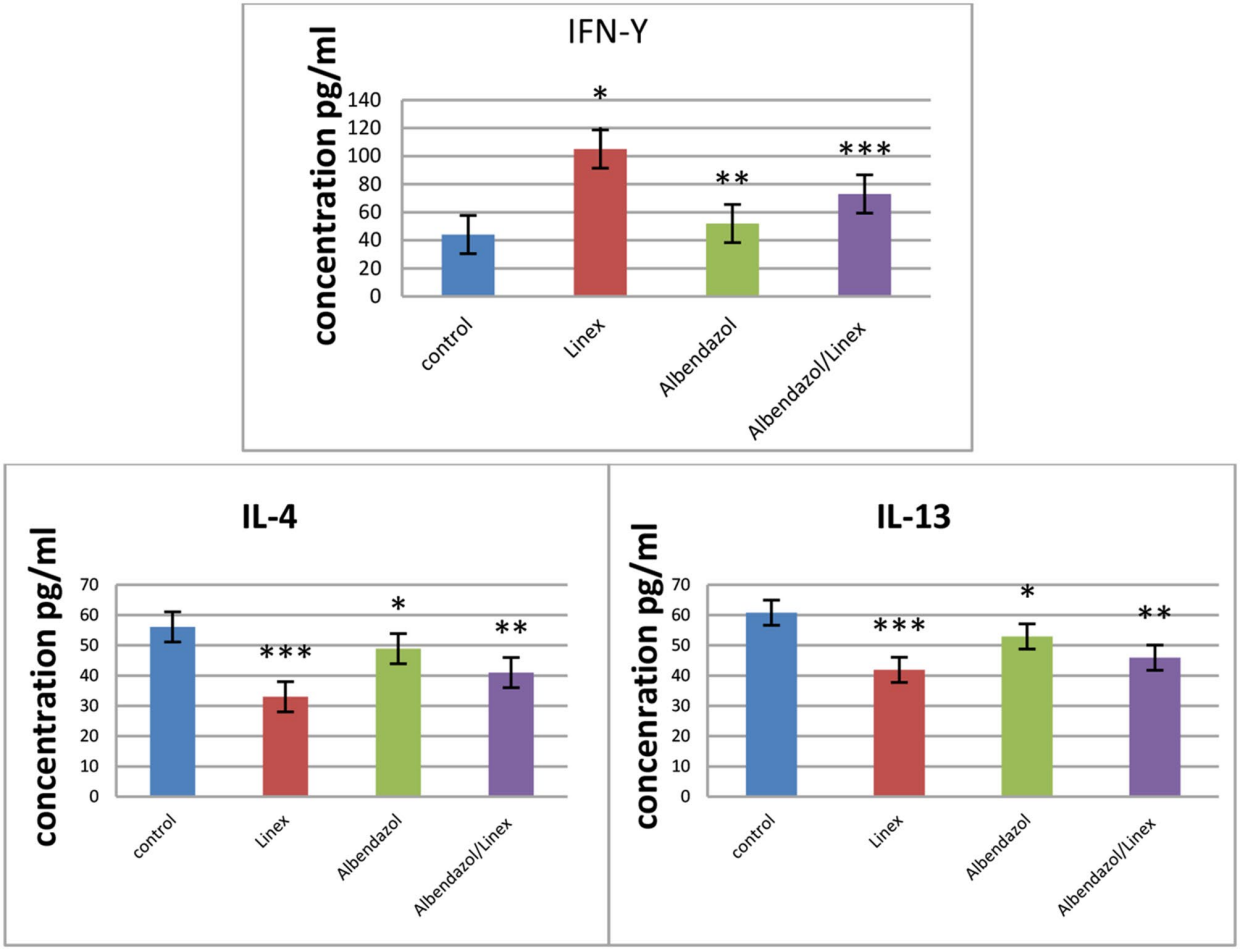
INF-γ, IL-4, Il-13 concentration in different groups

Immunological results with different treatnment modalities compared to infection control group *P* < 0.05 are summarized in Table 3; Fig. 5.

**Table 3:** INFγ, IL-4, Il-13 concentration in different groups expressed as mean ± SD

Groups	INFγ (pg/mL)	IL-4 (pg/mL)	IL-13 (pg/mL)
Normal control group	85.8 ± 28.5	30.0 ± 1.79	12.0
Infection control (IVa)	44.11 ± 0.107	56.10 ± 0.115	60.74 ± 0.526
Linex group (IVb)	51.95 ± 0.171*	33.16 ± 0.298***	41.92 ± 0.134***
Albendazole group (IVc)	73.03 ± 0.138**	47.61 ± 3.363*	52.95 ± 0.171*
Linex + Albendazole (IVd)	105.05 ± 0.096***	41.16 ± 0.472**	45.93 ± 0.149**

*p < 0.05: significant compared to infection control

**p < 0.05: significant compared to infection control

***p < 0.05: significant compared to infection control

### Overall therapeutic efficacy of different treatments groups

Generally there is improvement in parasitological, histopathological, and immunological parameters in all treated groups with most promising results in combined Albendazole and Linex therapy.

## Discussion

In experimentally infected mice, the therapeutic effect of commercially available probiotic (Linex) and Albendazole were tested individually and in combination on the intestinal and muscular stages of trichinellosis.

There was a significant reduction in both adult worm and larval burdens across all treated groups compared to the control groups, the percentage reduction in the number of adult worms and larvae in Albendazole treated group (IIIc, IVc) was 100% and 79.2%, respectively while the adult worm and larval load in group treated with Linex (IIIb, IVb) was 43.2% and 88.4%, respectively.

Our findings suggest that the combination of Linex and Albendazole offers a highly effective treatment strategy for Trichinella spiralis infection, with the group treated with combined Albendazole and Linex (IIId, IVd) showing a 100% reduction in the number of adult worms, and a 97.7% reduction in larval load, the synergistic effect of Linex may be attributed to its impact on the immune response against Trichinella spiralis as emphasized by Dvorožňáková et al. [21], who found that the administration of probiotic strains enhances the phagocytosis and respiratory burst of blood PMNLs, potentially leading to decreased larval migration, destruction of muscle larvae, and subsequently reducing the parasite burden in the host in addition, it has been found to increase the superoxide activity of the macrophages which enhances the host defense against intestinal and muscular phase of infection [22]. In addition to Linex effect on the systemic immune response of the host it may also has a direct effect on the adult worm through affecting the local immune response as probiotics have the ability to compete with other pathogens for adhesion sites or by enhancing intestinal mucosal barrier activity [23, 24], Furthermore, probiotics play a major role through their interactions with the gut-associated lymphoid tissue (GALT), a key immune system component within the intestines. Probiotics influence various immune cells within GALT, such as enterocytes, dendritic cells, and T-cells, enhancing the body’s defense against intestinal pathogens. Additionally, the relationship between gut microbiota composition and nematode infections suggests that gastrointestinal bacteria play a significant role in the host’s immune response to parasitic infections [25].

During the last decade, several studies have emphasize the effect of probiotic on intestinal parasite control [26, 27]. Many of these studies support our findings and study the effect of different probiotic strains on Trichinella and other parasites where Naguib et al. [28] found that there is a 59.86% reduction in adult worms treated with lactoferrin alone in Trichinella spiralis infected mice. Another study by Bocktor et al. [29] found that the percent reduction in the larval load in mice treated by combination of *L. acidophilus* and Albendazole was 92.5% while with *L. acidophilus* and nitazoxanide was 56.62% with a significant difference when compared to a regimen involving a single drug administration either ABZ (78.26%) or NTZ (40.95%) therapy. In a simillar approach another study found that addition of probiotic to Ivermectin show 88.1% reduction in the larval load in Trichinella spiralis infected mice compared to Ivermectin alone with percent reduction of 79.6% in the larval load, and 38.2% using Probiotic alone [30].

Many other studies used Probiotic on different parasites as toxoplasmosis where [31] found that lactoferrin reduce parasite burden and affect the tachyzoite viability, another study of Moussa et al. [32] revealed that lactoferrin have a protective effect against toxoplasmosis. León-Sicairos and his colleagues [33] study the amoebicidal effect of apo-lactoferrin by killing the parasite trophozoite.

The role of probiotic in reducing inflammation has been explored especially in reducing gut and muscle inflammation [34, 35].

Our study showed that there has been a reduction in the inflammatory response in the intestinal and muscle stage of the experiment in all treated groups. Albendazole only treated group (IIIc, IVc) reveles inflammatory changes affecting the number and the integrity of Trichinella spiralis cyst, in muscle there is few cysts with focally degenerated capsule while the intestinal tissue of Albendazole treated group reveals a normal villous pattern, both muscle and intestinal tissue of this group show moderate inflammatory cellular infiltrate, if we compare this results with that of the Linex only treated group (IIIb, IVb) Linex is found that it doesn’t have direct effect on Trichinella spiralis cyst but it show the same effect as Albendazole in reducing inflammation that affect both muscle and intestinal tissue where it demonstrate moderate decrease in the inflammatory cellular infiltration of plasma cells and lymphocytes in intestinal and skeletal muscle sections, while combined Albendazole and Linex therapy show mild inflammatory cellular infiltrate and complete degeneration of Trichinella spiralis capsule and larvae this may be due to the strong Anti-inflammatory effect of Linex which potentiate the action of Albendazole, this agree with a study done by Bocktor, et al. [29] suggest that addition of Probiotic to Ivermectin and Nitazoxanide potentiate the effect of both drugs in alleviating the inflammatory response and reducing inflammatory cellular infiltration of lymphocytes, plasma cells and histiocytes infiltrating infected muscle fibers and per capsular area of encysted larvae.

Those results also have been emphasized in many studies where Li et al. [36] found that *Lactobacillus* and *Bifidobacteria* have strong antioxidant and anti-inflammatory effect in alleviating inflammatory bowel disease, many studies postulate the mechanism by which Probiotic can reduce inflammation in the affected organ where it binds to receptors on host cells followed by induction of secretion of anti-inflammatory compounds by the host which result finally in decreasing of inflammatory response and reducing cell damage [37, 38]. Probiotics also can stimulate the intestinal immune system by activating the aryl hydrocarbon receptor (AhR) pathway, a key regulator of inflammation [39]. Some studies explain the ability of probiotics in supporting intestinal epithelial cell regeneration and inhibition of apoptosis through Toll-like receptors (TLRs), like TLR-2 and TLR-4, leading to the production of protective cytokines [40]. Certain lactic acid bacteria strains can produce acetylcholine, which is found that it helps in reducing mucosal intestinal inflammation [41].

The effect of probiotic on reducing muscle fiber inflammation as present in our study may be attributed to the immunomodulatory effect of probiotics.

The significant results of Linex especially when added to Albendazole suggest that it may has immunomodulatory effect on Trichinella spiralis infection also some studies demonstrate that Probiotic may has immunomodulatory effect against several pathogens and degenerative disease [8], affecting both innate and adaptive immune responses [42].

Our results showed increased in IFN-γ levels and decreased IL-4 and IL-13 with treatment with Linex, Albendazole or both during the muscular phase of infection.

It is documented that Trichinella infection release immunoregulatory products that predominantly induce Th2 immune responses. During the intestinal phase, the immune response initially exhibits a mixed profile, with a predominant Th1 response characterized by increased interferon gamma (IFN-γ), interleukin-2 (IL-2), and interleukin-12 (IL-12). Subsequently, there is an elevation in the Th2 response, characterized by increased interleukin-4 (IL-4), IL-5, IL-9, IL-10, and IL-13 [43, 44]. However in the muscular phase, with establishment of the immunomodulatory effect of Trichinella, the pattern of cytokines changes. This immune polarization involves the production of cytokines as IL-4, IL-5, IL-9, IL-10, IL-13, IL-21, and IL-33 [45–48], and on the other hand is associated with decrease in some cytokines as IFN γ [49].

Cytokines such as IFN-γ, play pivotal roles in the immune response against Trichinella spiralis. Upon T cell stimulation, activated macrophages produce IFN-γ, which demonstrate robust parasite-killing properties [50, 51]. Differential expression patterns of cytokines like IFN-γ occur during different stages of infection, highlighting the dynamic immune response during trichinellosis [44, 52]. IFN-γ enhances the cytotoxicity of various immune cells against Trichinella spiralis larvae [51].

IL-4 has been shown to be important in the development of protective responses to gastrointestinal nematode infections; exogenous IL-4 treatment has been shown to reduce fecundity and cure established Heligmosomoides polygyrus infection in BALB/c mice and Nippostrongylus brasiliensis in the highly susceptible severe combined immunodeficient (SCID) mouse [53, 54].

Both IL-4/IL-13 protect against Trichinella spiralis by activating signal transducer and activator of transcription 6 (Stat6), Stat6 activation protects against this parasite through different mechanisms. Stat6- signaling promotes immunity to Trichinella spiralis both through effects on bone marrow-derived cells and through effects on non-bone marrow-derived cells. The former effects appear to include T-cell-dependent induction of intestinal mastocytosis, while the latter sensitize non-bone marrow-derived cells to mast cell-produced mediators [55].

Despite functional similarities to IL-4, IL-13 has been shown to mediate its effects independently of IL-4. IL-13 is shown to stimulate fibroblasts and lead to the development of fibrosis [55, 56].

Our cytokine analysis further reinforces the parasitological and histopathological results. The combined therapy induced a significant increase in IFN-γ levels (105.05 pg/mL), indicating a strong Th1 immune response. Meanwhile, IL-4 and IL-13 levels were significantly reduced, suggesting a shift away from the Th2-dominated immune response typically seen during chronic parasitic infections.

Many studies target shifting immune response from Th2 to Th1 immune response to control Trichinella spiralis as Del Prete et al. [57] who demonstrate that The Helicobacter pylori neutrophil-activating protein (HP-NAP) enhances endogenous IL-12 and IFN-γ response and exerts a powerful anti-T(H)2 activity in vivo, targeting both IL-5-induced eosinophilia and IL-4-mediated hyper-IgE responses induced by Trichinella spiralis infection.

Increase in the number of CD8^+^ cells and a decrease in the number of CD4^+^ cells, have been observed in individuals with general immune activation caused by persistent helminthic infection [58].

Our result show that Linex treatment has the most significant immunmodulatory effect in shifting immune response toward Th1 where IL-4 and IL-13 show significant decrease to 33.16 pg/mL and 41.92 pg/mL respectively compared to infection control group 56.10 pg/mL and 60.74 pg/mL respectively, and so addition of Linex to Albendazole enhance the immune response against muscular phase of Trichinella than Albendazole effect alone.

Similarly, combined Albendazole and Linex therapy show the most promising results in enhancing Th1 response where the level of IFN-γ increase to 105.05 pg/mL in compared to infection control group 44.11 pg/mL.

Albendazole is the mainstay treatment for Trichinellosis and has marked impact on the immune profile during parasitic infections [59], yet its effectiveness against encapsulated larvae is restricted, where some studies demonstrate failure of Mebendazole in killing encapsulated larva in mice muscle [60], so there is a raising concerns about drug resistance. Hence, exploring alternative anti-parasitic compounds, particularly from natural sources, is mandatory [61].

Probiotic confer immunological protection to the host through the regulation and modulation of immune responses.

In an in vitro study with CACO-2 cells by Haller and his colleagues [62] found that *Lactobacillus sakei* and *Lactobacillus johnsonii* increase the production anti-inflammatory cytokines (TGF-*β*), other anti-inflammatory cytokines released by Th2 cells, DCs, B cells in response to Probiotic administration are IL-4, IL-5, IL-6, IL-10, and IL-13, inducing an adaptive immune response in the body [63] Several studies demonstrate the effect of different strains of Probiotics on down regulation of proinflammatory cytokine early in the disease as INF-*γ* and TNF-*α*, while later on in the disease during muscular phase TH2 cytokines predominate and INF-*γ* increase [64].

This study emphasize that we can use this commercially available Linex drug which is used safely by patients, in combination with Albendazole so, a potential reduction in the dosage or duration of Albendazole therapy could take place which could minimize its known adverse effects, also this combination could offer a novel approach in decreasing Albendazole resistance by improving the drug efficacy and potentiation of the immune response, Further studies are needed to clinically validate these combination and study different drug dosage and duration are needed.

## Data Availability

The raw data of this work from which statistical analysis was done is available.

## Abbreviations

ALB: Albendazole
IL: Interleukin
INFγ: Interferon gamma
OD: Optical density
PMNLs: Polymorph nuclear leukocytes
T.S.: Trichinella spiralis
